# Spermidine activates RIP1 deubiquitination to inhibit TNF-α-induced NF-κB/p65 signaling pathway in osteoarthritis

**DOI:** 10.1038/s41419-020-2710-y

**Published:** 2020-07-06

**Authors:** Zhong Chen, Chuang-Xin Lin, Bin Song, Chang-Chuan Li, Jun-Xiong Qiu, Shi-Xun Li, Si-Peng Lin, Wen-Qiang Luo, Yuan Fu, Gui-Bin Fang, Li Wei-Ping, Phei Er Saw, Yue Ding

**Affiliations:** 1 Department of Orthopedics, Sun Yat-sen Memorial Hospital, Sun Yat-Sen University, Guangzhou, 510120 P. R. China; 2 Guangdong Provincial Key Laboratory of Malignant Tumor Epigenetics and Gene Regulation, Biomedical Research Center, Sun Yat-sen Memorial Hospital, Sun Yat-Sen University, Guangzhou, 510120 P. R. China; 3 Department of Orthopedic Surgery, Shantou Central Hospital, Affiliated Shantou Hospital of Sun Yat-Sen University, Shantou, 515000 P. R. China

**Keywords:** Nuclear receptors, Target identification, Drug development

## Abstract

Spermidine has been known to inhibit the production of pro-inflammatory cytokines. However, there are no reports about anti-inflammatory effects of spermidine on osteoarthritis (OA). Herein, we examined whether OA progression could be delayed by intraperitoneal injection (i.p.) of spermidine in the anterior cruciate ligament transection (ACLT) and TNF-α induced arthritis (TIA) mouse models. During the process, human FLS cells (H-FLS) were used to investigate the potential ubiquitination mechanism of spermidine-mediated RIP1 in TNF-α-induced NF-κB/p65 signaling. We found that spermidine attenuated synovitis, cartilage degeneration and osteophyte formation, resulting in substantially lower OARSI scores and TNF-α scores in spermidine-treated ACLT and TIA mice. In terms of the mechanism, 9 μM spermidine did not affect the viability, proliferation, cell cycle and apoptosis of H-FLS, and exerted inhibitory effects by activating CYLD-mediated RIP1 deubiquitination on TNF-α-induced NF-κB/p65 signaling in H-FLS. From these data, we can conclude that spermidine attenuates OA progression by the inhibition of TNF-α-induced NF-κB pathway via the deubiquitination of RIP1 in FLS. Therefore, intake of spermidine could be a potential therapy for preventing OA.

## Introduction

Osteoarthritis (OA) is one of the most common form of arthritis and is the leading cause of joint pain and disability^1^. OA is a disease of complex pathological mechanism that could be caused by various factors, including age, obesity, joint trauma, and instability^2^. However, due to the inherent complexity of biological factors that consists of joint loading, aging, obesity with metabolic syndrome and inflammation^3^, current treatments which cannot prevent the progression of OA, leading to sustained structural damage of the knee, are deemed ineffective.

During the onset of OA, the most common symptom is synovial inflammation of the diseased joint^4^. Inflamed joints bring about increasing pro-inflammatory cytokines such as TNF-α^5^ produced by fibroblast-like synoviocytes (FLS). Previous studies have shown that TNF-α is highly expressed in synovial tissue of osteoarthritic joints^6–8^. In the pro-inflammatory cascade, TNF-α would ultimately activate the most important NF-κB/p65 transcriptional pathways, which in turn secretes IL-6 and IL-8 leading to the aggravation of OA^9,10^. However, until now, the greatest drawback for anti-TNF-α agents is its potent systemic toxicity, which poses some potential safety issues, including infection and autoimmune diseases^11^. In light of this, nutraceuticals and naturally occurring compounds represents another safe opportunity for anti-TNF-α treatment of OA.

The natural polyamine spermidine was reported to have cardioprotective effects, dietary spermidine can reduce TNF-α in plasma^12^ and further increase the bioavailability of NO through the reduction of oxidative stress in cardiomyocytes^13^. In another study, intraperitoneal injection (i.p.) of spermidine analog, spermine, reduces TNF-α in the plasma of septic mice via the inhibition of high mobility group protein-1 (HMGB1)-induced release of inflammatory cytokines in macrophages^14^. However, there are no reports on whether spermidine could inhibit TNF-α-induced inflammation in OA. Herein, the focus of our research is to explore the role of spermidine in TNF-α-induced inflammation in OA, determine safe and effective drug concentrations, and explain its specific location and molecular targets, which could provide a safe and reliable alternative treatment for OA.

## Methods

### Materials

TNF-α (Catalog #: ab6671), Collagen 10 (Catalog #: ab49945), MMP3 (Catalog #: ab52915), MMP13 (Catalog #: ab39012), iNOS (Catalog #: ab15323), UBA1 (Catalog #: ab34711) Adamts4 (Catalog #: ab1855722) and Adamts5 (Catalog #: ab41037) were purchased from Abcam (MA, USA), Adalimumab (Catalog #: A2010) and Glucosamine (Catalog #: S6400) were purchased from Selleckchem (TX, USA), UBE2N (Catalog #: 6999), phospho-RIP1 (Catalog #: 65746), NF-κB phospho-p65 (Catalog #: 3033), NF-κB p65 (Catalog #: 8242), RIP1 (Catalog #: 3943), Anti-rabbit IgG (H + L) Alexa Fluor 555 (Catalog #: 4413) and Anti-mouse IgG (H + L) Alexa Fluor 488 (Catalog #: 4408) were purchased from Cell Signaling Technology (MA, USA), Vimentin (Catalog #: SC6260), HA-Tag (Catalog #: SC7392), IκB (Catalog #: SC1643) and phospho-IκB (Catalog #: SC8404) were purchased from Santa Cruz Biotechnology (CA, USA), Spermidine (Catalog #: 05292) and Aggrecan (Catalog #: AB1031) were purchased from Sigma (MO, USA), CLYD (Catalog #: 1110-1-AP) and TRIM21 (Catalog #: 12108-1-AP) were purchased from PeproTech (NJ, USA).

### Arthritis mouse model and spermidine treatment

#### PTOA mouse model and spermidine treatment

Male C57BL/6J mice aged 12 weeks were purchased from the Experimental Animal Centre of SiBeiFu (SPF Biotechnology Co. Ltd., Beijing, China) and housed in a pathogen-free animal facility at the Sun Yat-Sen University. Then the mice were randomly divided into two main groups (*n* = 150): (1) ACLT operation group (*n* = 100), where mice underwent anterior cruciate ligament transection (ACLT) surgery to destabilize the joint and induce post-traumatic OA (PTOA), (2) sham operation group (*n* = 50), where a sham operation was performed with a similar incision at the right joint capsule. On the next day, ACLT and sham groups were further administered with: (i) phosphate-buffered saline (PBS), (ii) 0.3 mM spermidine, (iii) 3 mM spermidine, (iv) 6 mM spermidine, and (v) 100 mg/kg DMSO, i.p. injection per day. Mice were euthanized for articular cartilage, synovial tissue (infrapatellar area), and osteophyte analysis after 4 weeks or 8 weeks of treatment.

#### TIA mouse model and spermidine treatment

The C57BL/6J mice were treated with TNF-α (10 μg/μl/week) by intra-articular injection to stimulate TNF-α-induced arthritis (TIA). The specific operation was as follows: TNF-α was injected into both knee cavities. Then the mice were randomly divided into three groups (*n* = 15): (i) control group, (ii) DMSO treatment group, or (iii) spermidine treatment group. For each treatment, the mice were further divided into three subgroups: (i) 4 weeks treatment (*n* = 5), (ii) 8 weeks treatment (*n* = 5), and (iii) 16 weeks treatment (*n* = 5). After respective treatments, the mice were euthanized and their knees were harvested for TNF-α immunohistochemistry analysis.

All animal experiments were approved by the Sun Yat-sen University Animal Ethics Committee (L102012016080F) and were performed in accordance with the Committee’s guidelines.

### Micro-CT

After the knee specimens were isolated, the surrounding soft tissues were removed and fixed with 4% PFA for 48 h. Micro-CT scan was performed for the knees at 100 kV, 98 μA, 12 μm resolution on a Viva CT40 Micro CT Scanner (ScancoMedi-cal AG, Bassersdorf, Switzerland). The entire joint was chosen as the region of interest (ROI) for three-dimensional (3D) reconstruction, osteophyte score and the volume of ROI were analyzed.

### Hematoxylin and eosin (H&E) and Safranin O-fast green staining

The knee joints were subjected to formalin fixation and was then decalcified with 0.5 M EDTA at pH 8.0 for 4 weeks, and was subsequently dehydrated and embedded in paraffin. The tissue sections were cut at 4–6 μm thickness in a sagittal orientation. H&E and Safranin O-Fast Green staining was performed, and the OARSI OA cartilage histopathology assessment system was chosen to assess the cartilage degeneration severity as described^15^.

### Immunohistochemical staining

For immunostaining, the sections were deparaffinized, briefly washed with 0.1 M PBS (pH 7.4), and incubated for 10 min in 3% H_2_O_2_ to quench endogenous peroxidase activity. Primary antibodies were applied overnight at 4 °C or incubated for 2 h at 37 °C. After being washed three times with PBS and incubated with goat-anti-rabbit HRP-conjugated secondary antibody for 1 h at 37 °C, an immunohistochemical staining signal was developed with 3, 3′-diamino-benzidine (DAB, BOSTER Biological Technology, Wuhan, China). The numbers of positive cells and relative intensity were counted using Image J software (National Institutes of Health, MD, USA).

### Cell preparation

#### Mouse FLS

Synovial tissues were obtained from sham and PTOA mice and incubated overnight in PBS with 1 mg/mL Collagenase I (Roche Pharmaceuticals, Basel, Switzerland) at 37 °C for 5–6 h to isolate normal synoviocytes and OA–FLS (normal-FLS and OA–FLS), respectively.

#### Human FLS

Human FLS (H-FLS) were taken from patients with no osteoarthritic characteristics who underwent arthroscopic meniscus repair surgery. H-FLS were used between passages 4 and 9, as previously described^16^. This study was approved by the Medical Ethics Committee of Sun Yat-sen Memorial Hospital (SYSEC-KY-KS-158).

#### Mouse OA–chondrocytes

Mouse OA–chondrocytes were obtained from cartilage, briefly, cartilage tissue was obtained from the knees of PTOA mice. Dissected cartilage pieces were incubated overnight in PBS with 1 mg/mL Collagenase D (Roche Pharmaceuticals, Basel, Switzerland) at 37 °C for 5–6 h. The solution was then centrifuged at 1000 rpm for 5 min to discard the supernatant, the remaining precipitate that contains primary OA–chondrocytes was mixed with 5 mL medium and was then inoculated in a culture flask.

#### Chondrogenic cell line ATDC5

Since the procedure of obtaining normal cartilage from patients were not approved by the Medical Ethics Committee of Sun Yat-sen Memorial Hospital, we opted to use a chondrogenic cell line (ATDC5) purchased from Riken BioResource Center (Tsukuba, Japan). The cells were induced into normal chondrocytes by the addition of insulin, transferrin, and selenous acid (ITS, Sigma-Aldrich) according to previous studies^17^.

### Quantitative real-time PCR

Cells were collected by scraping with 1 mL of PBS and centrifuged for 10 min at 3000 rpm to collect cell pellets, and total RNA was isolated from cell pellets using TRIzol reagent (Life Technologies, NY, USA). cDNA was transcribed from RNA samples by using reverse transcription reagents (Sangong Biotech Co. Ltd., Shanghai, China) and quantitative real-time polymerase chain reaction (qRT-PCR) assays were carried out to quantify the levels of mRNA expression of these genes. GAPDH was used as the internal loading control using qRT-PCR Mix (Proteintech, IL, USA) and the Light Cycler (Roche Switzerland). All primer sequences used in this manuscript are listed in Supplementary Table 1.

### Enzyme-linked immunosorbent assay (ELISA)

The TNF-α, IL-6, IL-8, and IL-4 level of inflammatory cytokines in serum and culture medium were measured using ELISA kits (R&D Systems, USA) according to the manufacturer’s instructions.

In order to identify the effective concentration of spermidine on TNF-α in OA–FLS, OA–FLS cells were treated with 3, 6, 9, 12, and 15 μM of spermidine, or 7 nM adalimumab or 5 nM glucosamine for 24 h. In our experiments, adalimumab^18^ was used as the positive control drug for TNF-α pathways. Glucosamine showed no anti-TNF-α activity in OA, we used glucosamine as a negative control.

### CCK-8 viability assay

Half-maximal inhibitory concentration (IC_50_) is the most widely used and informative measure of a drug’s efficacy, where less than 5% cell activity inhibition rate is the safe range of the drug^19^. According to the protocol of the drug IC_50_ experiment, the viability of cells were measured by the cell counting kit-8 (CCK-8, Dojindo, Japan), then, IC_1_, IC_3_, and IC_5_ were obtained.

### EdU proliferation assay

The effects of spermidine, adalimumab or glucosamine on the proliferation of H-FLS were measured by 5-ethynyl-2ywdeoxyuridine (EdU) assay. EdU staining was conducted using a Cell-Light EdU DNA Cell Proliferation Kit (RiboBio, Guangzhou, China) according to the manufacturer’s protocol and images were captured using an Olympus laser scanning microscope system (Olympus FV3000, Japan).

### Cell-cycle analysis and apoptosis assay

Briefly, 1 × 10^5^ cells per well were seeded into 6-well culture plates and incubated with either spermidine, adalimumab, or glucosamine for 24 h. Cell-cycle and Apoptosis experiment was conducted according to the manufacturer’s protocol (BD Biosciences, San Diego, USA). Cell-cycle and apoptosis were quantified using the FACS Caliber flow cytometer (BD, NJ, USA).

### Immunofluorescence staining

H-FLS cells were treated with TNF-α (10 ng/mL) in the presence or absence of spermidine (9 μM). After 1 h, cells were immersed in 4% paraformaldehyde (PFA) for 15 min. The cells were treated with 0.1% Triton-100 for permeabilization and blocked in 1% bovine serum albumin in PBS for 30 min, followed by incubation with primary antibodies overnight at 4 °C. The cells were then followed by incubation with appropriate secondary immunofluorescent antibodies for 60 min. After washing three times with PBS, cells were added with Anti-fade Fluorescence Mounting Medium with DAPI (Guangzhou HelixGen Co., Ltd., China), and were imaged by confocal microscopy (Zeiss LSM 710, Germany).

### Lentivirus transfection in FLS for CYLD knockdown

The shRNA-CYLD and GFP that were packaged as lentivirus were purchased from Gene Copoeia (MD, USA). H-FLS was divided into two groups (i) shRNA-CYLD group and (ii) shRNA-Scrambled Control (shRNA-SC) group. Transfection efficiency of >80% was considered successful and the cells were then used for subsequent experiments.

### RIP1 ubiquitination assays

Plasmids were constructed for RIP1 ubiquitination overexpression. Briefly, the coding regions of the genes were amplified from the Homo lung cDNA library via the PCR technique. Then the resulting fragments were cut with restriction enzymes, and inserted into pcDNA3.1-Myc and pcDNA3.1-HA-Ubiquitin (Invitrogen) to form pcDNA3.1-RIP1(WT)-myc and pcDNA3.1(+)-HA-Ub (RIP1 plasmid and HA-Ub plasmid) for overexpressing RIP1(WT) gene and ubiquitination, respectively. All constructs were sequenced for confirmation.

To determine the role of spermidine effects on RIP1 ubiquitination, the RIP1 plasmid and HA-Ub plasmid were co-transfected overexpression of RIP1 (WT) and ubiquitinated plasmid (2 μg/μL) for 48 h. The transfection efficiency was determined by using Western blot analysis.

To determine the role of spermidine effects on RIP1 deubiquitination by the activation of CLYD, H-FLS cells were divided into two groups: (i) shRNA-CYLD group and (ii) shRNA-SC group. Both groups were then co-transfected with RIP1 (WT) and ubiquitinated plasmid. After transfection of shRNA-CYLD lentivirus and RIP1 (WT) and HA-Ub plasmid, the transfected H-FLS cells were stimulated with 10 ng/mL of TNF-α for 2 min in the presence or absence of 9 μM spermidine for 1 h, then, cells were resuspended in 1 mL of cold PBS, and were subsequently directly lysed by RIPA lysis buffer (50 mM Tris, pH 7.4, 150 mM NaCl, 1 mM EDTA, 20 mM N-ethylmaleimide and 1% Triton X-100). The lysate was immediately incubated with anti-RIP1 and A/G Plus Agarose beads. Ubiquitinated RIP1 proteins were detected by immunoblotting with an HA antibody and were subsequently probed with anti-RIP1 as a loading control.

### Western Blotting analyses

In the experiment of inflammation signaling pathway of H-FLS, H-FLS cells were treated with TNF-α with or without spermidine for 24 h, cells lysates were then separated by SDS-PAGE and transferred to PVDF membranes. Membranes were blotted with primary antibodies recognizing Adamts4, iNOS, MMP3, MMP13, NF-κB p65, NF-κB phospho-p65, IκBα, phospho-IκBα, RIP, phospho-RIP1, CYLD, Iκκ-β, and GAPDH, respectively. All blots were probed with horseradish peroxidase (HRP)-conjugated secondary antibodies (Cell Signaling Technology, USA), and immunoreactive proteins were revealed using the enhanced chemiluminescence (Millipore, USA) to detect the targeted proteins.

The Western Blot membrane was scanned (G: BOX Gel & Blot Imaging Series from Syngene, UK) and calculated the numerical intensity by Image J 4.5 analysis system (National Institutes of Health, MD, USA). Intensity of each protein was normalized with GAPDH and was represented as a ratio to the control.

### Statistical analyses

Data are presented as mean ± standard deviation (mean ± S.D). Differences between two groups were statistically analyzed by unpaired, two-tailed Student’s *t* test. Differences among three groups were analyzed by one-way analysis of variance (ANOVA) and Dunnett’s multiple comparison tests. The level of significance was set at *p < 0.05, **p < 0.01, ***p < 0.001. All statistical analyses were performed with GraphPad Prism software version 7.0 (GraphPad Software, Inc., CA, USA).

## Results

### Spermidine attenuates progression of OA in PTOA mouse models

**Fig. 1:**
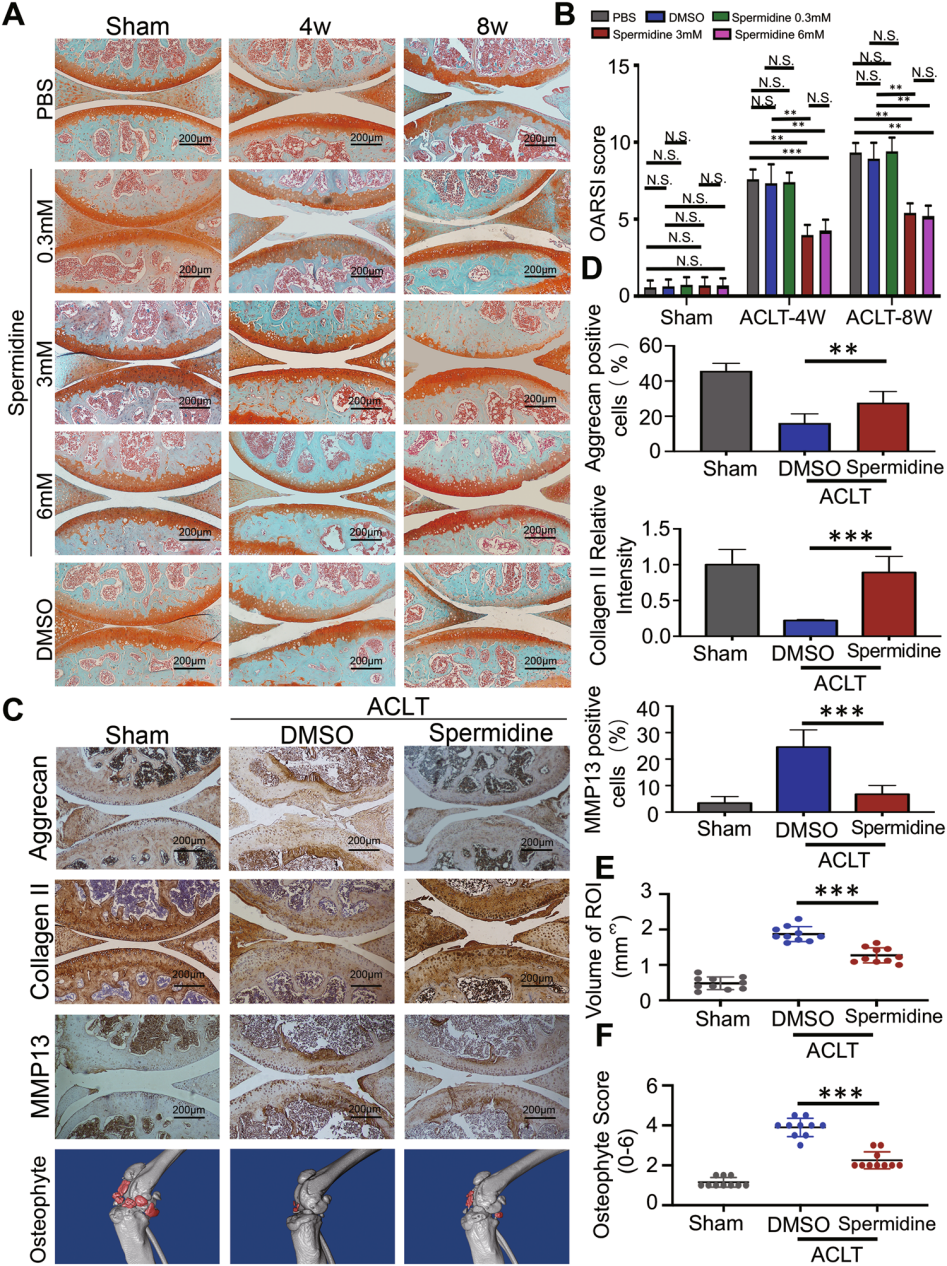
Spermidine treatment ameliorates articular cartilage degeneration and osteophyte in the ACLT mouse models. **a** Safranin-O-fast green staining of the medial tibial plateau joint of wild-type mice at 4 and 8 weeks after surgery. **b** Quantitative analysis of OARSI score (whole joint). **c** Immunohistochemistry showing Aggrecan, Collagen II and MMP13 expression in articular cartilage and micro CT scan, 3D reconstruction of the knee joint from Sham + DMSO, ACLT + DMSO, and ACLT + spermidine-treated mice at 8 weeks after ACLT surgery. **d** Quantitative analysis of Aggrecan- and MMP13-positive cells and Collagen II relative intensity in articular cartilage. **e** Quantitative analysis of osteophyte score and volume of region of interest (ROI). The ROI is marked in red for periarticular osteophytes. Data are shown as mean ± SD, *n* = 10, *p < 0.05, **p < 0.01, ***p < 0.001, scale bar, 200 µm.

Safranin O staining demonstrated retention of proteoglycan and decreased thickness of calcified cartilage zone in 3 or 6 mM spermidine-treated ACLT mice relative to 0.3 mM spermidine-, DMSO-, and PBS-treated ACLT controls both at 4 and 8 weeks (Fig. 1a), OARSI scores were significantly reduced in 3 or 6 mM spermidine treated ACLT mice relative to 0.3 mM spermidine, DMSO and PBS-treated ACLT mice both at 4 weeks (*p* < 0.01) and 8 weeks (*p* < 0.01), OARSI scores were not significantly different between 3 mM spermidine- and 6 mM spermidine-treated ACLT mice (4 weeks *p* = 0.6486; 8 weeks, *p* = 0.9576) (Fig. 1b). Besides, spermidine significantly increased the expression of Aggrecan and Collagen II, and reduced the expression of MMP13 as assessed by immunostaining in spermidine-treated ACLT mice relative to DMSO-treated ACLT mice at 8 weeks (*p* < 0.01) (Fig. 1c, d).

Compared to the sham group, at 8 weeks post operation, DMSO-treated ACLT mice developed larger periarticular osteophytes with significantly increased volume and surface area of osteophytes (Fig. 1e). However, with the treatment of spermidine, the volume and surface area of osteophytes around the joint were significantly reduced in ACLT mice (*p* < 0.001) (Fig. 1f). These findings suggested that spermidine treatment could help ameliorate cartilage degeneration and osteophyte formation during OA progression in PTOA mouse models.

### Spermidine inhibits synovial inflammation-mediated cartilage degeneration in PTOA mouse models

To further investigate the effects of spermidine on cartilage degeneration, primary OA–chondrocytes were isolated in ACLT mice at 8 weeks post operation. Then we treated cultured primary chondrocytes with IC_1_ = 3 μM, IC_3_ = 6 μM, and IC_5_ = 9 μM drug concentration of spermidine (IC_50_ = 102.557 μM) (Fig. S1A) at different time points to examine whether spermidine has a biological effect on the degeneration and hypertrophic differentiation of primary OA–chondrocytes. The expression of Aggrecan, Collagen10, Adamts4, Adamts5, MMP3, and MMP13 were not significantly different in spermidine treatment groups compared to the blank group, independent of either the drug concentration or duration; as determined by qRT-PCR (Fig. S1B) and Western blot analysis (Fig. S1C, D). These results indicate that spermidine may not have a biological activity against chondrocyte degeneration and terminal differentiation.

**Fig. 2:**
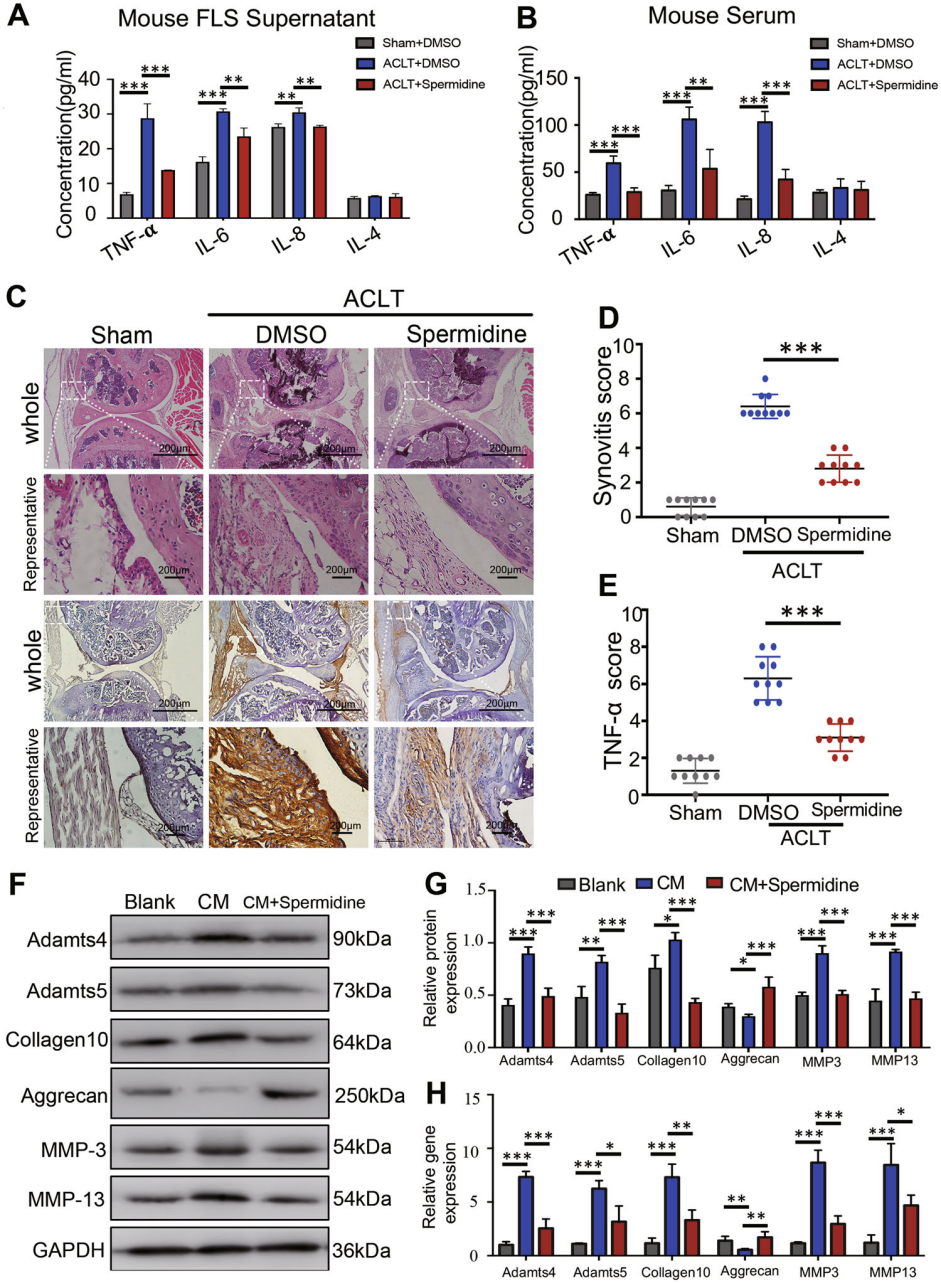
Spermidine treatment reduces the synovial inflammation-mediated cartilage degeneration. **a**, **b** Inflammatory cytokines TNF-α, IL-6, IL-8, and IL-4 were measured in FLS supernatant or serum from ACLT mice using ELISA. **c** Hematoxylin-eosin (HE) staining (above) and immunohistochemistry (IHC) results (below) of the medial tibial plateau joint of Sham, ACLT + DMSO, ACLT + spermidine mice 8 weeks after ACLT, IHC using antibodies against TNF-α. **d** Synovial inflammation score was calculated based on HE staining. **e** TNF-α score was calculated based on IHC staining. **f** Western blot and **g** quantification of protein expression and (**h**) qRT-PCR analysis of Adamts4, Adamts5, Aggrecan, MMP3, MMP13, and Collagen10 expression of ADTC5 chondrocytes treated with the supernatant of DMSO-treated primary OA–FLS (CM) or spermidine-treated primary OA–FLS (CM + spermidine) for 24 h. Data are shown as mean ± SD, *n* = 10, *p < 0.05, **p < 0.01, ***p < 0.001, scale bar, 200 µm.

However, through ELISA analysis, we found that spermidine treatment could significantly reduce the levels of TNF-α, IL-6, and IL-8 in the supernatant of cultured primary OA–FLS of ACLT mice (Fig. 2a), which have been demonstrated to be increased in synovial tissue during PTOA progression^20,21^. Consistently, the levels of serum TNF-α, IL-6, and IL-8 were also significantly reduced in spermidine-treated ACLT mice compared to DMSO-treated ACLT mice at 8 weeks (Fig. 2b). Consistent with previous results^22^, intra-articular synovial hyperplasia and abundant cell infiltration were observed in the DMSO-treated ACLT mice at 8 weeks (Fig. 2c), which result in significantly higher synovitis scores than that of the sham controls (*p* < 0.001) (Fig. 2d). We discovered that spermidine administration by i.p. significantly reduced TNF-α expression in synovial tissue in spermidine-treated ACLT mice compared to DMSO-treated ACLT mice at 8 weeks (*p* < 0.001) (Fig. 2c, e). These findings demonstrated that spermidine treatment could inhibit synovial inflammation in PTOA mice models.

To elucidate the effect of spermidine on synovial inflammation-mediated cartilage degeneration, ADTC5 cells were induced by selenous acid and then co-cultured with the supernatant of DMSO-treated primary OA–FLS (conditioned medium, CM) or spermidine-treated primary OA–FLS (CM + spermidine) for 24 h. Western blot and qRT-PCR analysis showed the expression of Aggrecan, Collagen10, Adamts4, Adamts5, MMP3, and MMP13 were significantly reduced in the spermidine-treated primary OA–FLS compared to DMSO-treated primary OA–FLS (Fig. 2f–h). Taken together, these findings demonstrated that spermidine treatment may inhibit synovial inflammation-mediated degeneration of articular cartilage in OA.

### Spermidine inhibits TNF-α-induced synovial inflammation in TIA mouse models

**Fig. 3:**
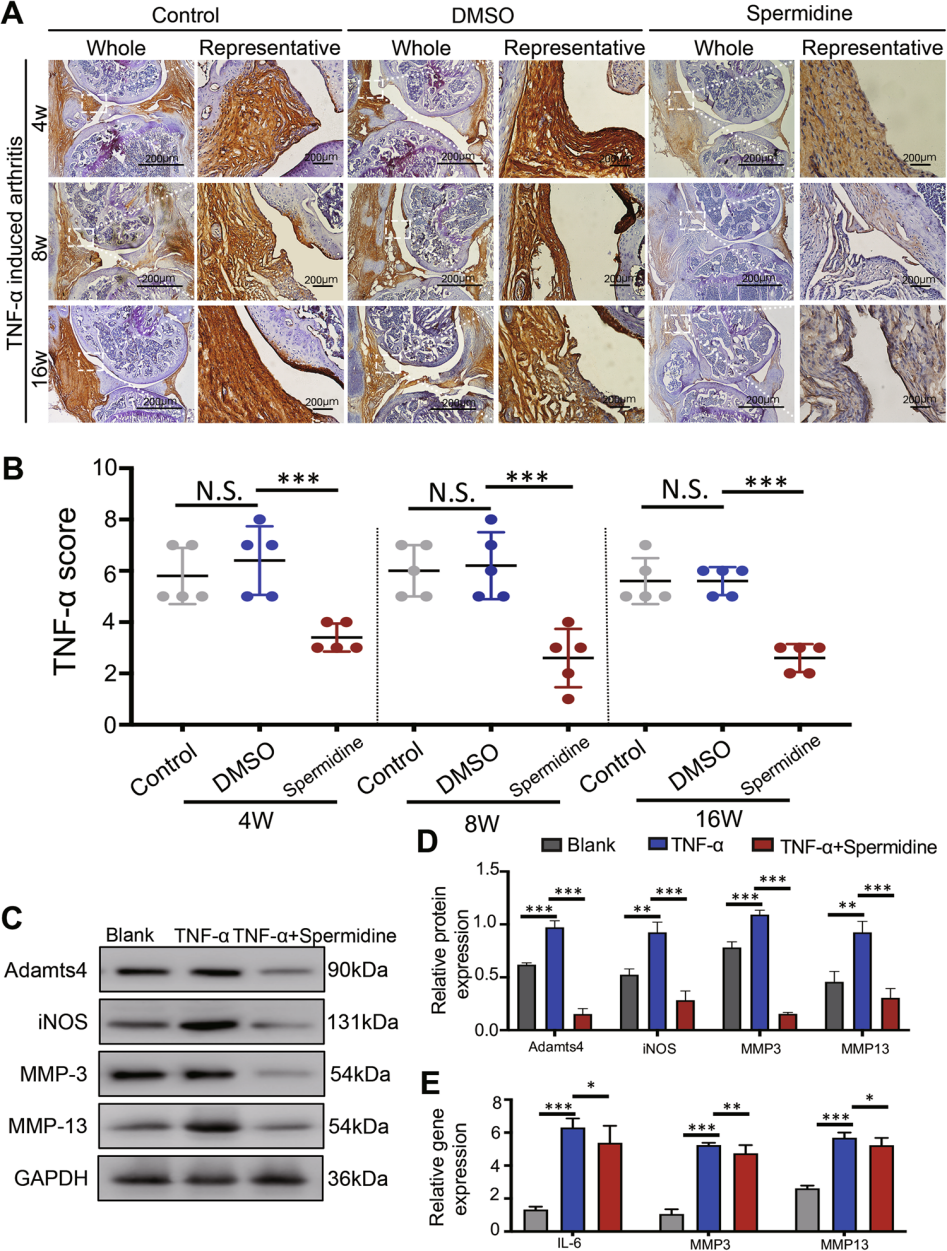
Spermidine treatment inhibits TNF-a induced arthritis (TIA). **a** Immunohistochemistry was used to assess the expression of TNF-α in synovial tissue of the knee joint after 4, 8, and 16 weeks of spermidine treatment to TNF-a induced arthritis mice. **b** TNF-α score was calculated based on IHC staining. **c**, **d** Western blot analysis of the expression of iNOS, MMP-3, MMP-13, and Adamts4 in FLS. **e** qRT-PCR analysis of the expression of IL-6, MMP-3, and MMP-13 in FLS. Data are shown as mean ± SD, *n* = 10, *p < 0.05, **p < 0.01, ***p < 0.001, scale bar, 200 µm.

Since synovial arthritis in OA is mainly due to the response to TNF-α production^23^, the IC_50_ of Mouse-OA–FLS and H-FLS were determined by CCK-8 assay (Fig. S2A, B),we then tested whether the safety of IC_1_, IC_3_, and IC_5_ (range from 3 to 12 μM) drug concentration of spermidine inhibited the production of TNF-α. ELISA analysis showed that spermidine treatment significantly reduced the production of TNF-α, where 9 μM of spermidine had the same anti-TNF-α effect as adalimumab (7 nM) in mouse-OA–FLS (*p* = 0.998) and TNF-α stimulated H-FLS (*p* = 0.999) (Fig. S2A). In addition, applied with a spermidine concentration of 12 and 15 μM, H-FLS cell apoptosis rate also was significantly increased (Fig. S3C, D). Here, we determined that 9 μM spermidine was effective and displayed a safe drug concentration for subsequent experiments of H-FLS. The expression of TNF-α in the synovial tissue of the spermidine-treated groups was significantly lower than that in the DMSO-treated groups (Fig. 3a). The TNF-α score of the synovial tissue of the treatment group was significant reduced compared with the DMSO group at 4 weeks, 8 weeks, or 16 weeks after treatment (Fig. 3b). In addition, the expression of pro-inflammatory factors IL-6, iNOS, metal matrix degrading enzymes MMP3, MMP13, and Adamts4 in TNF-α and spermidine-co-treated FLS was significantly lower than that of TNF-α-treated FLS (Fig. 3c–e). These findings confirm that spermidine treatment could inhibit TNF-a-mediated synovial inflammation in vivo and in vitro.

### Spermidine prevents TNF-α-induced NF-κB/p65 activation by suppressing RIP1 ubiquitination

**Fig. 4:**
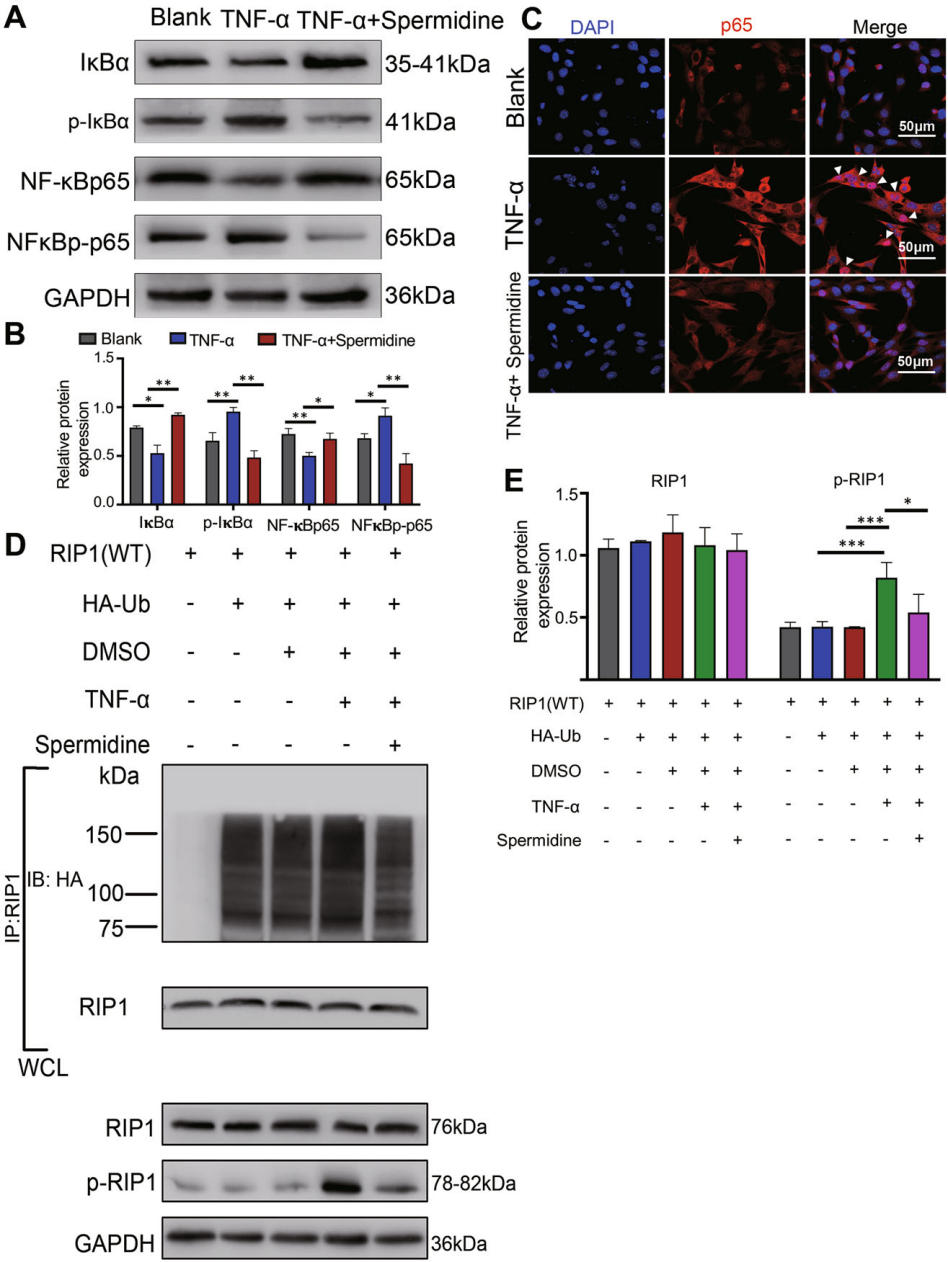
Spermidine inhibits TNF-α induced NF-κB/p65 inflammatory signaling by inhibiting RIP1 ubiquitination in FLS. **a** Immunoblot analysis and **b** quantification of protein expression of phosphorylated (p-) or total protein IκBα and p65 of H-FLSs treated with TNF-α (10 ng/mL) and spermidine (9 μM) for 24 h. **c** Immunofluorescence analysis of p65 (white arrow) activation and translocation into the nucleus of FLS; scale bar, 50 μm. **d** RIP1 ubiquitination, RIP1 and phosphorylation(p-) of RIP1 was assessed by immunoprecipitation and immunoblotting and **e** the relative protein expression in H-FLS co-transfected with RIP1(WT) and HA-Ub plasmid, and treated with TNF-α (10 ng/mL) and spermidine (9 μM). Data are shown as mean ± SD, *n* = 3, *p < 0.05, **p < 0.01, ***p < 0.001, scale bar, 200 µm.

The expression of NF-κB/p65 and IκBα were significantly increased, while the phosphorylation of NF-κB/p65 (NF-κB/p-p65) and IκBα (p-IκBα) were significantly reduced in TNF-α and spermidine-treated H-FLS than that of TNF-α-treated H-FLS alone, which indicated that spermidine treatment inhibited TNF-α-induced NF-κB/p65 activation (Fig. 4a, b). Immunofluorescence assay identified that TNF-α stimulation increased NF-κB/p65 activation and translocation into the nucleus of FLS (Fig. 4c, white arrow) and the translocation of NF-κB/p65 was significantly reduced after spermidine treatment (Fig. 4c). These results suggested that spermidine treatment could inhibit TNF-α-induced NF-κB/p65 activation in H-FLS. As shown in Fig. 4d, e, RIP1 ubiquitination and phosphorylation were significantly increased in the TNF-α-stimulated group compared with the DMSO-treated group (Fig. 4d [lane 3 vs. lane 4]), and spermidine treatment inhibited TNF-α-induced RIP1 ubiquitination and phosphorylation (Fig. 4d [lane 4 vs. lane 5]), and, as we expected, RIP1 ubiquitination was not detected by Western blot in non-transfected H-FLS (control). (Fig. 4d [lane 1]). These results indicated that spermidine treatment was likely to inhibit the activation of TNF-α-induced NF-κB/p65 pathway by inhibiting RIP1 ubiquitination.

### Spermidine upregulate CYLD to activate RIP1 deubiquitination

**Fig. 5:**
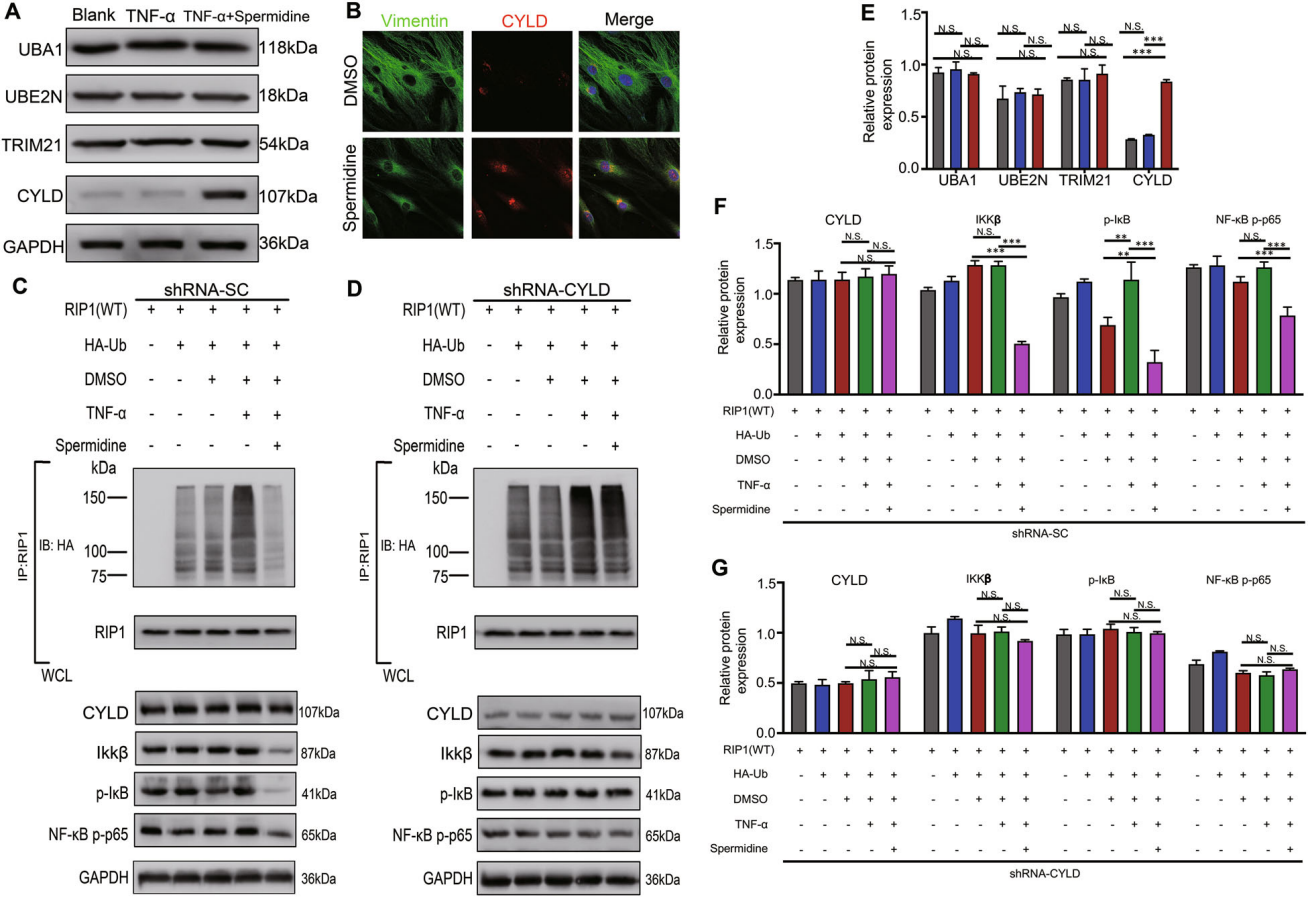
Spermidine actives CYLD to prompt RIP1 deubiquitination. **a** Immunoblot analysis of the expression of UBA1 (E1), UBE2N (E2), TRIM21 (E3), CYLD in H-FLS treated by TNF-α for 2 min and spermidine for 1 h. **b** Immunofluorescence analysis of CYLD expression on the cytoplasm in H-FLS; scale bar, 20 μm. **c** RIP1 Ubiquitination, RIP1, CYLD, IKKβ, phosphorylated IKKβ (p-IKKβ), phosphorylated p65 (p-p65) was assessed by immunoprecipitation and immunoblotting in H-FLS transfected with HA-Ub and RIP1 (WT) plasmid, shRNA-Scramble (shRNA-SC) and treated with TNF-α (10 ng/mL) and spermidine (9 μM). **d** RIP1 Ubiquitination, RIP1, CYLD, IKKβ, phosphorylated IKKβ (p-IKKβ), phosphorylated p65 (p-p65) was assessed by immunoprecipitation and immunoblotting in H-FLS transfected with HA-Ub and RIP1 (WT) plasmid, shRNA-CYLD1 and treated with TNF-α (10 ng/mL) and spermidine (9 μM). **e**–**g** Quantification of protein expression for (**a**), (**c**), and (**d**) analysis of immunoprecipitation and immunoblotting respectively. Data are shown as mean ± SD, *n* = 3, *p < 0.05, **p < 0.01, ***p < 0.001, scale bar, 20 µm.

In order to determine which enzyme was activated by spermidine treatment to suppress the RIP1 ubiquitination in H-FLS, (E1)-UBA1, (E2)-UBE2N, (E3)-TRIM21, and CYLD enzyme were detected by Western blot after H-FLS were treated by TNF-α for 2 min and spermidine treatment for 1 h. The result showed that spermidine treatment significantly increased the expression of CYLD enzyme compared to the absence of treatment or TNF-α treatment (Fig. 5a, e). This result was further confirmed by cell immunofluorescence experiments in which spermidine treatment increased CYLD expression in the cytoplasm of H-FLS (Fig. 5b).

We generated two independently stable sh-CYLD-expressing FLS cells for further analysis and after transfection, the expression of CYLD protein was lower in sh-CYLD1-transfected H-FLS cells (Fig. S4A, B), and RIP1 protein levels were significantly increased in CYLD-depleted FLS cells (Fig. S4A, B). CYLD mRNA levels were lower in sh-CYLD1-transfected FLS cells than sh-CYLD2 and sh-SC (*p* < 0.0001), whereas RIP1 mRNA levels were unaffected (*p* > 0.05) (Fig. S4C). We found that spermidine treatment downregulated the ubiquitination of RIP1 and its downstream phosphorylation of IKKβ and p65 compared to TNF-α stimulation in FLS without silencing CYLD (Fig. 5c [lane 5 vs. lane 4], f). We also found that with silencing of CYLD in FLS, the ubiquitination of RIP1 was upregulated after TNF-α stimulation compared to control and DMSO treatment (Fig. 5d [lane 4 vs. lane 2 and 3], g). Importantly, spermidine treatment failed to downregulated ubiquitination of RIP1 (Fig. 5d [lane 5 vs. lane 4], g). Together, these results suggest that spermidine prompted the deubiquitination of RIP1 by upregulating CYLD in TNF-α-induced NF-κB activation.

## Discussion

In this study, we found that i.p. administration of spermidine could effectively reduce the expression of TNF-α in FLS and serum in OA mice model and inhibit the deubiquitination of RIP1 in TNF-α-mediated NF-κB/p65 activation, by activating CYLD in FLS. Taken together, all these resulted in significant inhibition of synovial inflammation, while cartilage degeneration and osteophyte formation were significantly ameliorated (Fig. 1), indicating the extension of previous findings for the ability of spermidine to reduce TNF-α mediated NF-κB/p65 signaling pathway in FLS.

As contrasted with RA, there are clear drug guidelines for anti-rheumatic drugs for anti-RA treatment^24^. However, spermidine analog putrescine was highly expressed in RA–FLS compared with OA–FLS^25^, and polyamine metabolism were positively contributes to RA-FLS intrinsic activation^26^. These results indicated that the polyamine metabolism process is the target of RA inhibition^27^, and spermidine supplementation may not work for RA. So far, it has been reported that oral intake of spermidine could reduce the expression of TNF-α in plasma^12,14,28^, which indicated a potential association between spermidine and anti-TNF-α inflammation. The theory of TNF-α-induced RIP1 ubiquitination signal of NF-κB/p65 pathway leading to inflammatory response in OA–FLS has been widely confirmed^23^. RIP1 ubiquitination displayed important recruitment and activation of the IKK complex that improved NF-κB activation^10^. Loss of RIP1 ubiquitination allows RIP1 to bind caspase-8, and thereby activates apoptosis^9^. Based on the above results, we speculate that the ubiquitination of RIP1 may determine whether its downstream product could activate NF-κB or apoptosis signaling. Interestingly, spermidine could prevent or reduce the apoptotic response triggered by TNF-α stimuli in non-tumoral cells^29–31^. Therefore, we estimate that RIP1 ubiquitination independent of apoptosis may be an important target for spermidine against TNF-α-mediated inflammation, and our results also confirmed the role of spermidine in RIP1 deubiquitination in TNF-α-driven NF-κB activation (Fig. 4d). This seems contradictory, considering spermidine induces the reduction of RIP1 ubiquitination but does not lead to cell apoptosis. Actually, ubiquitinated RIP1 has undergone protein modification, which reduces the action in the apoptotic pathway, conversely, the absence of RIP1 ubiquitylation will mediate the apoptotic pathway because cell membrane surface receptors such as T cell Receptor (TcR)and Toll-Like Receptor 4 (TLR-4) and DNA damage are activated and integrated through RIP1 activation of downstream apoptosis and necrosis signals^32^. However, spermidine is also a double-edged sword, which could also activate apoptosis^33^. In our results, increased apoptosis of H-FLS cells was found when spermidine concentration was greater than 12 μM (Fig. S2). Therefore, when using spermidine to activate RIP1 deubiquitination, it is necessary to precisely adjust the concentration of spermidine to avoid activation of the apoptotic pathway of RIP1 in H-FLS cells.

**Fig. 6:**
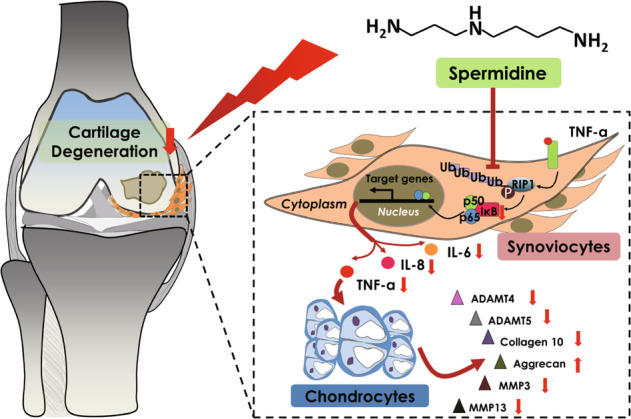
Spermidine inhibit the ubiquitination of RIP1 in TNF-α driven NF-κB/p65 inflammation pathway in Fibroblast synoviocytes (FLS) of osteoarthritis (OA). The RIP1 ubiquitination that inhibited by spermidine resulting in the decreased phosphorylation of the IKK complex including the most important p65 transcription pathways. Afterward, the inflammatory cytokines such as IL-6, IL-8 and TNF-a, were decreasingly secreted by FLS. The decreased infiltration of inflammatory cytokines on cartilage, which ameliorate the degenerative expression of chondrocytes including Aggrecan, Collagen10, ADAMTS4, ADAMTS5, MMP3 and MMP13, and finally alleviate the progression of OA.

Accumulating evidence highlights the vital role of deubiquitinating enzymes in the pathogenesis and progression of arthritis^34–36^. CYLD is a NEMO interacting protein that removes the polyubiquitin chain linked to the K63 position of RIP1, thereby inhibiting IKK and NF-κB activity^37,38^. However, previous studies have not applied a safe stimulant to activate the endogenous CYLD deubiquitination system that effects the target protein of RIP1, which is not suitable for clinical application. Importantly, in this study, CYLD could be enhanced by spermidine and promoted the deubiquitination of RIP1 in TNF-α-induced NF-κB activation (Fig. 5c, d). Notably, CYLD mediates RIP1 deubiquitination though removal of the Iys63 polyubiquitin chain, allowing RIP1 to bind to Caspase 8 and Caspase l0 to promote cell apoptosis^39,40^. It seems that the ubiquitination of RIP1 protein by CYLD still via apoptosis signaling. We did not detect apoptosis after overexpression of the CYLD gene in H-FLS, because the non-apoptotic dosage of spermidine in cytotoxicity experiments have been determined. In summary, spermidine deubiquitinates RIP1 in TNF-α-induced NF-κB activation by upregulating CYLD, thereby reducing synovial inflammation and cartilage degeneration (Fig. 6).

## Conclusion

In summary, our study provides compelling evidence supporting the role of ubiquitination-regulated inflammation of spermidine in the treatment of OA. The therapeutic effect of spermidine on OA is dependent on FLS rather than direct regulation of chondrocytes. This work suggests that spermidine could be a promising drug candidate for OA intervention.

## Supplementary information

Supplementary Table 1

Figure S1

Figure S2

Figure S3

Figure S4
