# Supplementary material for: Spermidine activates RIP1 deubiquitination to inhibit TNF-α-induced NF-κB/p65 signaling pathway in osteoarthritis
Source: Cell Death Dis. 2020 Jul 6;11(7):503. doi: 10.1038/s41419-020-2710-y (PMC7338517; doi:10.1038/s41419-020-2710-y)
Supplement: Supplementary file 1 — Supplementary Table 1 [file 41419_2020_2710_MOESM1_ESM.docx]

**Supplementary Table 1. Primer sequences for qRT-PCR**

| **Gene (Species)** | **Forward primer** | **Reverse primer** | |
| --- | --- | --- | --- |
| Adamts4  (Mouse) | GACTGGCACCATCAATGGAGA | CTTCCGGCGTAGGATGTGAG |  |
| Adamts5  (Mouse) | GGAGCGAGGCCATTTACAAC | CGTAGACAAGGTAGCCCACTTT |  |
| Collagen10  (Mouse) | TTCTGCTGCTAATGTTCTTGACC | GGGATGAAGTATTGTGTCTTGGG |  |
| Aggrecan  (Mouse) | CCTGCTACTTCATCGACCCC | AGATGCTGTTGACTCGAACCT |  |
| MMP-3  (Mouse) | TTAAAGACAGGCACTTTTGGC | CCCTCGTATAGCCCAGAACT |  |
| MMP-13  (Mouse) | CTTCTTCTTGTTGAGCTGGACTC | CTGTGGAGGTCACTGTAGACT |  |
| IL-6  (Mouse) | CTTCTTCTTGTTGAGCTGGACTC | \| AAGTGCATCATCGTTGTTCATACA \| \| --- \| |  |
| CYLD  (Human) | AAGGCTACAGGATCTACCTCAG | GTGGTTGTGAGTCAACAGAAGA |  |
| RIP1  (Human) | TTACATGGAAAAGGCGTGATACA | AGGTCTGCGATCTTAATGTGGA |  |
| GAPDH | AAATGGTGAAGGTCGGTGTGAAC | CAACAATCTCCACTTTGCCACTG |  |
